# The New York Institute of Stomatology

**Published:** 1897-09

**Authors:** 

**Affiliations:** The New York Institute of Stomatology


### THE NEW YORK INSTITUTE OF STOMATOLOGY.

A REGULAR meeting of the Institute was held Tuesday evening, May 4, 1897, at the residence of Dr. S. E. Davenport, No. 51 West Forty-seventh Street, New York City. The President, Dr. George Allan, occupied the chair.

*Dr. G. A. Wilson.*—I have here two volumes,—the report of the State Board of Health of Connecticut for 1895 and 1896,—forwarded to us by Dr. George L. Parmele, recorder of Dental Commissioners of Hartford. I have extended the thanks of the Institute to Dr. Parmele.

COMMUNICATIONS ON THEORY AND PRACTICE.

*Dr. J. F. P. Hodson.*—I have the pleasure of presenting to the Institute a little device which has recently become of very great value to me. I have never used any of the usual matrices that have been offered to the profession, for the reason, among others, that they are chiefly but variations of a straight band around the tooth, which produces in the finished stopping a convexity in only one direction, and in its longitudinal aspect a surface as literally flat as if a file had been run between the teeth, and having little or no real adjustment to the cervical edge of the cavity. I want the rotundity to be a real and complete one. There is just as great necessity for the convexity to obtain from the grinding surface to the cervical as from the buccal to the lingual. Some years ago I conceived this little appliance, and have used it since so continuously that it has come to be quite as important as my rubber dam, or anything that I depend upon for aid in my daily practice. These little matrices are simple, delicate, easily made and applied, and, while doing their duty well, are so little offence to the patient that they may be—and should, for amalgam—left in place until the next sitting. The material is very thin steel. (I happen to find a very suitable sort in that which the women employ for the bottoms of their gowns.) After annealing slightly, cut it with scissors to the different sizes and general shapes of the approximal aspect of the tooth, pass a file around all its edges to smooth it for the tongue, and also to prevent any burred edges interfering with its slipping to place, punch a tiny hole near the buccal and lingual edges for convenience of removal, then place the strip on a piece of lead and strike with the hammer a small convex bead held upon it (I use a small, round-headed picture-nail, which I case-hardened and polished), thus stretching (not merely bending) the thin steel into a concave condition in every direction. It is quite easy to give in a moment just the convexity needed for the individual case, and it is sometimes of value, in irregularly placed teeth, to convex it more on one part of the approximal surface than another. The delicate strips will go into a very thin space and hug the surface like a glove, being coaxed to their places with any large end pusher, and with a curving motion, presuming, of course, the surfaces to have received the usual polishing preparatory to filling. The convexity that impinges against the adjoining tooth, combined with the tempering effects of the hammer-taps in forming them, gives to them sufficient strength and stiffness to hold their place against the tooth perfectly. They are very firm and strong. For amalgam stoppings I leave them in place over night, and, upon taking them out the next day, have a shaped surface that is ideal.

I sometimes use them for gold stoppings, in which case the buccal and lingual edges, being somewhat free, allows them to give very slightly and produce the little overplus that we require for the final burnish condensation of edges, which is, of course, done at once, and finally finished before dam-removal. With the amalgam, however, this does not obtain, the edges being already solid. Indeed, in the latter case, having at the subsequent sitting taken away the little matrix, that slips out beautifully, there is usually nothing to do but to polish the perfectly convexed stopping, and it is finished.

I have here three or four teeth lashed together and partly invested, in a hurried endeavor to approximate the natural resiliency of the teeth in their sockets. A solid investment, not being the mouth condition, would show nothing of the delicate way in which the teeth in the mouth give to let in these tiny but powerful strips. I have filled one of the teeth with amalgam and taken out the matrix, leaving the filling untouched, merely turning the matrix to the opposite cavity in the same space (which, by the way, I should *always* leave to be filled until after one side was *finished*). It will be noticed that the cervical edge of that filling is absolutely perfect; there is no overplus to be removed, except the slight, usual one at the top near the grinding surface.

*Dr. Elliott.*—Does Dr. Hodson make a special matrix for each case?

*Dr. Hodson.*—I have a dozen or two on hand in order to save time, and can usually select from them, but it takes but a minute or two to make a new one, and so it is not of sufficient importance to worry me if I do not have one which answers the three essentials of (1) such size, bucco-lingually, as that it shall not be too long, and to keep its ends close to the tooth, that they may slip between the tooth and gum; (2) such width from grinding to cervical edge as that, upon being slipped over the cervical edge of the cavity, it shall not project above the grinding surface to be hit by the tooth in the other jaw; (3) such convexity as shall make it fit to its place perfectly "snug,"—no more, no less. The matrices come out, however, very easily, no matter how tight they go in; the delicate spring tightness of their adjustment, combined with the subsequent filling, makes a slight wedging, so that they slip out with perfect ease by means of two little excavators placed in the holes made in the edges for that purpose, and with the curving motion mentioned in first placing them; and this very convexity holds them securely from falling out before the patient is again seen.

*Dr. J. Morgan Howe.*—Dentists are often asked, "What is good for the toothache?" and they find it a difficult question to answer. I have brought with me something to-night that is good for the toothache,—some little sample-bottles of a medicine I have found useful; I will give a short description of it, and ask the gentlemen to try it and give reports later on. I have had it in my medicine-case for some time, but have only lately been getting facts in regard to it. It is called mentho-phenol, and was produced by William Schaeffer, M.D. He published its composition and method of preparation in the *Boston Medical and Surgical Journal*, vol. cxxxiv. p. 111. It is obtained by adding one part of phenol crystals to three parts of menthol and melting the substances. It is a fluid, of pale amber color and aromatic odor, very pungent in taste, but is not a caustic. It dissolves readily in alcohol, ether, chloroform, and most oils, and is a solvent of iodoform and aristol. High antiseptic properties are claimed for it, while it undoubtedly possesses decided analgesic qualities. Dr. Schaeffer suggests a solution of two drops to an ounce of water as a mouth-wash. He has treated successfully, with a three-per-cent. aqueous solution, phagedenic chancroid ulcers, mucous patches of a syphilitic character, and erysipelatous suppuration of glands.

I made some of this a number of months ago, and have found it especially valuable in applications to aching pulps. A few days ago I had a very marked instance of its value, when, on opening a molar tooth that proved to be the cause of an abscess on account of the pulp being devitalized, the tooth began to ache violently when it was uncovered. As soon as the pulp-cavity was well opened, the pain in the tooth was very severe; the contents of the bulbous portion of the pulp-chamber was entirely putrid, and the dentine was saturated with the putrid material, so that it emitted a strong odor on drilling. I soon discovered that the pulp in two of the root-canals was living, and in the other it was dead to the apex, causing a large abscess. The portion of living pulp was so much irritated that it caused intense pain. Two or three applications of various medicaments were made to the exposed pulp-tissue without mitigating the pain in the least, but this mentho-phenol caused the pain to subside almost at once. There is no doubt that it has wonderful analgesic properties. Whether it will be found beneficial in the dressing of root-canals, when the teeth are causing pain from pericemental inflammation, remains to be discovered.

*Dr. Hodson.*—What is Dr. Howe's treatment of just such conditions of partially destroyed pulps?

*Dr. Howe.*—My treatment would be to get the partially destroyed pulp out of the tooth as soon as possible; but if the patient is suffering very much, my first object is to allay the pain. Partially destroyed pulps are not always the active cause of great distress, and in such cases it is often possible to remove the portion that remains in the pulp-canal without special treatment.

*Dr. Hodson.*—I mean an upper third, for instance, of a pulp that is yet alive. How would Dr. Howe get rid of it?

*Dr. Howe.*—My purpose is to get it out of the root-canal as quickly as I can. If I can produce local anæsthesia to a sufficient extent to enable me to remove it, I do so at once.

*Dr. Brockway.*—If not, Dr. Howe would make an application of arsenical paste?

*Dr. Howe.*—Yes; I would use some arsenicated preparation that would destroy the vitality.

In the case I referred to the patient was so unnerved that I could not think of doing anything except relieve the pain; in other cases local anæsthesia can be effected so as to remove the remnant at once; and in still other conditions resort to devitalizers seems to be best.

*Dr. Hodson.*—Just this particular thing has been the most irritating point to me in all my practice,—what to do with the last third of those disreputable little pulps.

*Dr. F. Milton Smith.*—I have a little appliance that I would like to show. We had a paper at the last meeting by Dr. Brockway on cleaning teeth. Some of the gentlemen did not like the idea of using the engine-brush on the teeth in the mouth of more than one patient. I confess to a little prejudice on that score myself. I know of no way of cleansing one of those brushes after it has been used on teeth in a filthy condition that would satisfy me to have it used upon my own teeth at the next sitting. There have been little rubber cups made for this purpose. The last I heard, they were fifty cents a dozen, which made them rather expensive. I have here a little device which is not original. Dr. McNaughton had one at one of our meetings, but owing to his extreme diffidence he did not present it. I learned of it some years ago at the First District Dental Society. It is a mandrel made with a little shoulder so that a bit of rubber tubing of the proper size will slip over the mandrel, the shoulder preventing the tubing from going too far. It is placed in the engine and a piece of coarse sand-paper used to give the tubing a knife-edge. The tubing costs very little and carries the polishing powders satisfactorily.

Dr. Stebbins then showed an apparatus for sterilizing instruments and explained the same.

*Dr. R. O. Stebbins.*—The boiler to this sterilizer is made from one piece of metal. The water should be poured into this boiler to about half an inch in depth. Instruments with the points up are placed in the rack or holder, which sets in the boiler, resting on the depression about one inch from the bottom. A small hole is made through the knob of the dome-shaped cover to allow the steam to escape. Steam is generated in one or two minutes by placing a lighted alcohol-lamp or Bunsen burner under the boiler.

It is not only necessary to clean from the instruments visible filth, but to remove microscopic germs of disease that may be lurking on the instruments two or three inches from the points. When the instruments are thoroughly sterilized remove the rack. In a moment the heat of the instruments will dry them. Water may be heated in the cup attachment for use at the operating-chair. In the cover to the cup, which presents a concave surface, gutta-percha will remain soft for fifteen minutes, there being no outlets for the steam. This metal cover attached to the boiler makes the sterilizer applicable for surgeons' use.

*The President.*—Can a higher heat than 212° F. be obtained ?

*Dr. Stebbins.*—Yes; if one stops the escape of steam through the hole in the cover he can get 240° or 250°.

*Dr. S. E. Davenport.*—I should like the privilege of presenting what Dr. J. N. Davenport, of Northampton, Massachusetts, calls the "poor man's crown." I suppose he means by that a crown constructed of inexpensive materials and requiring comparatively little of the operator's time to prepare it for insertion. The materials of which the finished crown is composed are an ordinary rubber, plain tooth, German-silver wire hammered into any shape that the opening in the root requires, and the ordinary vulcanizing rubber of any color decided upon. The *modus operandi* is very simple. The root-canal being first prepared, a plain, rubber tooth is selected and ground to the gum. The end of the root is ground fairly smooth, but one peculiarity about the method is that, if the palatine or lingual portion of the root stands rather high and is fairly strong, it is not necessary to cut it to the gum, as for most other methods, it being possible to make the crown conform to any irregularity of the root. A piece of German-silver wire, of suitable size, is hammered on the anvil to the proper shape and fitted to the root-canal. The hammering gives additional stiffness, and makes the wire so rigid that there is no possibility of bending it with the ordinary force which comes upon a crown. One end of the German silver is then made jagged with the file and passed between the pins of the tooth, which are bent to hold it. Red, pink, or white vulcanizing rubber in small pieces is now packed upon the pins and over the wire with a warm instrument, and in sufficient quantity to form a shoulder which will cover the end of the root. This unfinished affair is then put into place, the pin going to its full extent into the root-canal, and the porcelain pressed to its proper position. Before it is put into place it is held over the spirit-lamp to soften the surface of the rubber, and the moistened thumb and finger will form the rubber by pressure accurately to the end of the root. It can be taken out, the surplus of soft rubber trimmed off, and put back again, half a dozen times, if necessary, within a few minutes, until everything seems right, when it is finally removed, invested in plaster in the flask, vulcanized, and finished. There are many ideas which can be applied to this method. It has been suggested by Dr. Davenport that wax instead of rubber could be used for the first fitting, particularly when two or three root-pins were to be used, and after everything was adjusted the waxed crown could be placed in the flask, the flask opened, the wax taken out, and rubber packed in. Then, too, bicuspids can easily be formed and made quite sightly by the use of white rubber for the inner cusp. These crowns should be secured to place with zinc phosphate.

*Dr. Bogue.*—Unless the gentlemen should want to put in just such a tooth as Dr. Davenport described, I would say that Dr. Herbst, of Bremen, showed me almost the same process, only, instead of rubber, he used tin as an attachment. He had his tooth ready to place in the mouth inside of half an hour. The wire for the pivot was placed in position, the cuspid tooth to be attached was placed in apposition, and attached to the wire with Stent's composition, which was then chilled with cold water from a syringe. The tooth and wire were then withdrawn, invested in plaster, and when that had set and been warmed pretty hot, tin was melted and poured in where the Stent had been. As soon as it was cold, it was filed down and polished, the tooth being set with phosphate.

*Dr. V. Parker.*—A few years ago, when I was at Little Falls, I

used to make a crown something like this, using amalgam scraps instead of rubber.

*The President.*—I have a little case here that I wish to explain before I pass it around. So far as I know, it illustrates something that is entirely new. In excavating the labial cavity of a cuspid tooth I found it impossible to locate the pulp-chamber. No broach would enter, and apparently the canal had disappeared. Continuing the excavation and pressing back the gum, I came upon a condition of affairs represented in this model. The pulp was ossified. That is common enough, of course, although in this case the patient was only about thirty years old; but I found that the decay, while it had attacked the normal dentine to a great extent, had not attacked the secondary dentine which had filled the pulp-chamber. If this fact has been heretofore noticed, I have no record of it; but it is plain and unmistakable in this case. I pointed out to Dr. Wilson at the time that the agents which had destroyed the dentine and enamel had failed to destroy the secondary dentine. I have no explanation to make, but I simply desire it to go on record.

The paper of the evening, entitled "Infection and Disinfectants," was then read by the author, Dr. Farquhar Ferguson.

The discussion was in part as follows:

*The President.*—We all know that in General Grant's case the epithelioma was supposed to have been produced by irritation from a tooth. In my own practice I have never met a case of that description.

*Dr. Howe.*—In regard to the question asked, I would say I have never seen a case of epithelioma that was unquestioned in its origin, as being due to the irritation of a tooth. I know the facts as stated by the President in regard to General Grant's case; but I have supposed that the idea that a tooth was a factor in irritating the tongue, and thus giving rise to the malignant process, was only a supposition.

If I may be allowed to continue, I would say a few words on the subject of the interesting presentation of infection and disinfectants that Dr. Ferguson has made for us. I consider it unfortunate that none of us, so far as I know, have been able to prepare specially to discuss this paper. I was impressed with the presentation of the dangers of infection, and the variety of means by which it can be brought about, so that the suggestion comes perhaps to most minds, what a wonder it is that we are any of us alive, with all these dangers coming at us in so many unavoidable ways! The probability is that we would not be alive, if it were not for certain antiseptic powers that we have within us that enable us to withstand and resist infection. In dealing with the subject of disinfectants or antiseptics, I was surprised that Dr. Ferguson did not refer to the idea that has been presented of late, that the serum of the blood contains a very powerful antiseptic. It has been said by Professor Vaughn, of Michigan University, that the antiseptic powers of the blood serum were far greater than that of bichloride of mercury. The relative value of disinfectants or antiseptics, in our experience, can hardly be disassociated from certain medicinal properties, and, in fact, I think I can divine in some expressions of Dr. Ferguson that he is a good deal of that opinion in regard to disinfectants generally; because he said he would rather trust to the experience and common sense of medical practitioners than he would to scientific investigation entirely. It seems to me that this is the point we must all fall back upon; that which in our experience combats the disease process, which results from introduction of pathogenic organisms, is not always the agent that has proved in the laboratory or in the test-tube to have the highest germicidal power. In fact, it may be an agent that has little or none of that power. Medicinal agents influencing the recuperative and resisting power of the tissues have a great deal to do with the question of the value of antiseptics. There seems little doubt that iodoform,—which I noticed was conspicuously absent from Dr. Ferguson's list—has very feeble antiseptic or disinfecting powers; but has nevertheless very valuable qualities, which aid living tissues to assist the action of pathogenic bacteria. This has been recently proved by Dr. Lomry, of Löwen University, in an interesting series of experiments on living animals. I do not feel competent to discuss off-hand the relative values of the different agents that Dr. Ferguson referred to; but I would call to mind some we have found valuable that he did not refer to, such as formaldehyde and electrozone. The latter is prepared as a proprietary article, and yet its chemical properties I think are pretty well known.

*The President.*—I have stopped using bichloride of mercury almost entirely. Some years ago, having a case of disease of the antrum, I began treating it with one-four-thousandth of the bichloride solution and reducing the strength finally to one-ten-thousandth. The case would not heal. I stopped using bichloride of mercury and used common salt, three per cent., with aseptic water, and the case got well rapidly. I also had another case of disease of the antrum where I used bichloride of mercury without success, so that at the present time I do not use it, as I think it has an injurious effect on the soft tissue.

*Dr. Bogue*—The paper has delighted me and has shown me much that I did not know. It was in the hope that those doors that Dr. Ferguson has opened a little would be opened wider that I listened with all attention. There was one point that I hoped he would enlarge upon, and that was in regard to the infection of phosphorous necrosis. He intimated that through defective teeth infection might come from various causes. I was waiting to hear what position he would give the dental pulp, which necessarily, in the case of match-workers who suffer from necrosis, is exposed, or, having been exposed, is either hypertrophied or dead or superficially ulcerated. He spoke to us, to be sure, of the sentinels which are on guard, and of the attacking agents which are constantly presenting themselves, and I live in hopes that in his next talk he will take up not only infection but asepsis, and show us the difference between an antiseptic and an aseptic condition,—between a deodorizer and a preventive of decay. I hope he will bring up the question in regard to essential oils, which perhaps prevent the passage of bacteria because of their viscidity. Perhaps there is something more than that, but that idea came to me very forcibly.

*Dr. Smith.*—I am greatly delighted with Dr. Ferguson's paper. One of the points that specially impressed me was that he would rather accept the judgment of men who are familiar with the treatment and care of certain diseases than the bare experiments of the scientist. It is refreshing to hear one who is eminently qualified to deal with those things from a scientific stand-point give us that suggestion, for it seems to me oftentimes the idea is lost sight of, by our scientific friends, that the surroundings with which we work are not the same as they deal with. We know certain remedies will have certain effects, if they are surrounded by certain conditions, whereas, if the conditions are different, they have not those effects.

Another thought that occurred to me was in reference to the amount of heat considered necessary to destroy the different forms of bacteria. Boiling water is commonly thought sufficient to sterilize our instruments if we boil them long enough.

*Dr. Howe.*—In regard to essential oils, I think there is no question that some of them have very decided germicidal properties. Dr. Black has established that, and some medical writers have referred to them. The oil of cinnamon, notably, I think, has been credited with very high germicidal powers.

Adjourned.

S. E. Davenport, D.D.S., M.D.S.,
*Editor of The New York Institute of Stomatology.*

